# G_q_-Mediated Arrhythmogenic Signaling Promotes Atrial Fibrillation

**DOI:** 10.3390/biomedicines11020526

**Published:** 2023-02-11

**Authors:** Felix Hohendanner, Ashok Prabhu, Nicola Wilck, Verena Stangl, Burkert Pieske, Karl Stangl, Till F. Althoff

**Affiliations:** 1 Department of Cardiology and German Heart Center, Campus Virchow-Klinikum, Charité–University Medicine Berlin, Augustenburger Platz 1, 13353 Berlin, Germany; 2 DZHK (German Centre for Cardiovascular Research), Partner Site Berlin, 13316 Berlin, Germany; 3 Berlin Institute of Health at Charité–Universitätsmedizin Berlin, 10117 Berlin, Germany; 4 Max Delbrück Center for Molecular Medicine in the Helmholtz Association (MDC), 13125 Berlin, Germany; 5 Experimental and Clinical Research Center (ECRC), a Cooperation of Charité–Universitätsmedizin Berlin and Max Delbruck Center for Molecular Medicine (MDC), 13125 Berlin, Germany; 6 Department of Nephrology and Medical Intensive Care Medicine, Charité–Universitätsmedizin Berlin, 10117 Berlin, Germany; 7 Department of Cardiology and Angiology, Charité Campus Mitte, Charité–University Medicine Berlin, Charitéplatz 1, 10117 Berlin, Germany; 8 Arrhythmia Section, Cardiovascular Institute (ICCV), Hospital Clínic, Universitat de Barcelona, C/Villarroel N° 170, 08036 Barcelona, Spain; 9 Institut d’Investigacions Biomèdiques August Pi i Sunyer (IDIBAPS), 08036 Barcelona, Spain

**Keywords:** atrial fibrillation, G-protein signaling, IP_3_ receptors, arrhythmogenic Ca^2+^ release, biased ligands

## Abstract

Background: Atrial fibrillation (AF) is promoted by various stimuli like angiotensin II, endothelin-1, epinephrine/norepinephrine, vagal activation, or mechanical stress, all of which activate receptors coupled to G-proteins of the Gα_q_/Gα_11_-family (G_q_). Besides pro-fibrotic and pro-inflammatory effects, G_q_-mediated signaling induces inositol trisphosphate receptor (IP_3_R)-mediated intracellular Ca^2+^ mobilization related to delayed after-depolarisations and AF. However, direct evidence of arrhythmogenic G_q_-mediated signaling is absent. Methods and results: To define the role of G_q_ in AF, transgenic mice with tamoxifen-inducible, cardiomyocyte-specific Gα_q_/Gα_11_-deficiency (G_q_-KO) were created and exposed to intracardiac electrophysiological studies. Baseline electrophysiological properties, including heart rate, sinus node recovery time, and atrial as well as AV nodal effective refractory periods, were comparable in G_q_-KO and control mice. However, inducibility and mean duration of AF episodes were significantly reduced in G_q_-KO mice—both before and after vagal stimulation. To explore underlying mechanisms, left atrial cardiomyocytes were isolated from G_q_-KO and control mice and electrically stimulated to study Ca^2+^-mobilization during excitation–contraction coupling using confocal microscopy. Spontaneous arrhythmogenic Ca^2+^ waves and sarcoplasmic reticulum content-corrected Ca^2+^ sparks were less frequent in G_q_-KO mice. Interestingly, nuclear but not cytosolic Ca^2+^ transient amplitudes were significantly decreased in G_q_-KO mice. Conclusion: G_q_-signaling promotes arrhythmogenic atrial Ca^2+^-release and AF in mice. Targeting this pathway, ideally using G_q_-selective, biased receptor ligands, may be a promising approach for the treatment and prevention of AF. Importantly, the atrial-specific expression of the G_q_-effector IP_3_R confers atrial selectivity mitigating the risk of life-threatening ventricular pro-arrhythmic effects.

## 1. Introduction

Atrial fibrillation (AF) is the most common cardiac arrhythmia. As well as causing debilitating symptoms, AF is associated with considerable morbidity and increased mortality [1]. Current strategies to treat AF—both antiarrhythmic drugs as well as catheter ablation—are of moderate efficacy only. Moreover, while catheter ablation is associated with a certain procedural risk, antiarrhythmic drugs are generally not well tolerated and often have to be discontinued because of substantial side effects, including potentially life-threatening ventricular pro-arrhythmic effects [2].

Numerous underlying pathological entities and conditions promote AF through neurohumoral triggers [3]. Many of these stimuli activate receptors coupled to G-proteins of the G_q_-family (G_q_) defined by the α-subunit isoforms Gα_q_ and Gα_11_. In fact, pro-arrhythmic effects have been consistently demonstrated for the predominant cardiac G_q_-coupled receptors, namely the angiotensin II receptor type 1 (AT1 receptor), the endothelin-1 receptor A (ETA receptor), the M3 muscarinic acetylcholine receptor (M3 receptor) and the alpha-1 adrenergic receptor [4]. In addition, immediate as well as chronic responses to mechanical forces can promote arrhythmia [5]. Interestingly, we could recently demonstrate that G_q_ can form a functional mechanosignaling complex with Piezo1 [6]. However, even though abundant evidence points to a central role of G_q_-mediated signal transduction in AF, direct evidence of G_q_-mediated arrhythmogenic effects and the putative mechanisms is absent [4].

Ectopic activity, particularly within the pulmonary veins, can act as a trigger on a vulnerable atrial substrate and as a driver maintaining AF. Delayed after-depolarisations (DADs) constitute the most important mechanism of ectopic activity in AF. It has been shown that diastolic Ca^2+^ leak from the sarcoplasmic reticulum leads to an increased inward current via the Na^+^-Ca^2+^-exchanger is the underlying cause of DADs in patients with AF [7,8,9]. Ca^2+^ release from the sarcoplasmic reticulum is regulated by ryanodine receptors (RyR2) as well as a second set of Ca^2+^ release channels, the inositol 1,4,5-trisphosphate (IP_3_) receptors. A large body of evidence demonstrates that type 2 IP_3_ receptors (IP_3_R) facilitate arrhythmogenic Ca^2+^ leak and AF-related ectopic activity [10]. IP_3_R is activated by IP_3_ in response to G_q_-mediated signaling via phospholipase C. Of note, the expression and function of IP_3_R, but not of RyR2, are enhanced in AF [11]. From a pharmacological standpoint, it is most intriguing that IP_3_R expression in atrial myocytes is 6- to 10-fold higher than in ventricular myocytes and that IP_3_R-mediated electrophysiological effects on Ca^2+^ homeostasis are absent in ventricular myocytes [12,13]. As the use of all currently approved antiarrhythmic drugs is limited by potentially life-threatening ventricular pro-arrhythmic effects, this renders the G_q_-IP_3_R-signaling pathway a promising target for the treatment of AF.

Here we investigate possible arrhythmogenic mechanisms and effects of G_q_-mediated signaling in the context of AF, as well as its suitability as a pharmacological target.

## 2. Methods

### 2.1. Conditional Cardiomyocyte-Specific Gα_q_/Gα_11_-Deficient Mice

Mice with a tamoxifen-inducible, cardiomyocyte-specific Gα_q_/Gα_11_-deficiency (G_q_-KO) were kindly provided by Prof. Nina Wettschureck, Max-Planck-Institute for Heart and Lung Research, Bad Nauheim, Germany. Briefly, those Gq-KO mice harbor floxed Gnaq and Gna11^−/−^ alleles as well as a tamoxifen-inducible Cre recombinase under the promoter of the mouse αMHC (MYH6) gene (MHCCreERT2) as previously reported [14,15]. Cre-mediated recombination of floxed alleles was induced by intraperitoneal injection of 1 mg tamoxifen dissolved in 50 μL miglyol oil on 5 consecutive days in 8-week-old G_q_-KO mice. MHCCreERT2; Gnaq^WT/WT^; Gna11^+/+^ mice served as control group and likewise underwent the tamoxifen-induction protocol. Experiments were performed 2 weeks after the end of induction.

All animal experiments were approved by the responsible federal authority (LAGeSo Berlin, approval TVA G0006/18) and performed conforming to the guidelines from Directive 2010/63/EU of the European Parliament on the protection of animals used for scientific purposes. Reporting in the manuscript follows the recommendations in the ARRIVE guidelines.

### 2.2. Invasive Electrophysiological Studies in Mice

For electrophysiological studies, mice were anesthetized with isoflurane (1.6 vol.% isoflurane/air) and placed on a heated surgical pad to maintain a constant body temperature. Limb electrodes were inserted subcutaneously to record a 6-lead surface ECG. After hair removal, a midline cervical incision was made, and the right jugular vein exposed to introduce a 2-French Octapolar diagnostic catheter (CIBermouse cath; NuMed, Inc., Cross Roads, TX, USA) connected to a digital electrophysiology recording system (EP Tracer, CardioTek, Maastricht, The Netherlands). The distal tip of the catheter was positioned in the right ventricle in a way that enabled recording of ventricular electrograms with the distal electrodes and atrial electrograms with the proximal electrodes. Inducibility of AF was determined before and two minutes after intraperitoneal injection of 50 ng/g carbachol (Sigma-Aldrich) by programmed electrical stimulation according to a murine AF model previously described by Wakimoto et al. [16]. AF was defined as the occurrence of fragmented atrial electrograms with irregular cycle lengths below 25 ms and absolute ventricular arrhythmia for at least 1 s. Animals were subsequently euthanized with a lethal dose of isoflurane followed by cervical dislocation.

### 2.3. Animal In Vitro Experiments

All chemicals and reagents were obtained from Sigma-Aldrich (St Louis, MO, USA) unless noted otherwise. Tyrode solution contained (in mM): 130 NaCl, 4 KCl, 2 CaCl_2_, 1 MgCl_2_, 10 Dglucose, 10 Hepes; pH 7.4 with NaOH. Atrial cardiomyocytes were isolated from WT and G_q_-KO mice (n = 5 animals / group) as previously described using enzymatic digestion [17]. Cells were subsequently loaded with Fluo-4-AM (Thermo Fisher, Waltham, MA, USA), and [Ca^2+^]-related fluorescence was measured using confocal line-scan imaging (Zeiss LSM 800, excitation at 488 nm, emission collected at > 515 nm) [18]. Experiments were performed at 35°C in Tyrode solution (3 mM [Ca^2+^]), and Ca^2+^ transients were elicited using electrical field stimulation (1 Hz). Longitudinal scan lines were chosen, and the cellular nucleus visually identified and included when feasible. Line scan images were used to derive cytosolic and nuclear Ca^2+^ transient release and removal characteristics (i.e., peak fluorescence: F/F_0_; TF50: time to 50% of maximal Ca^2+^ release; TAU: decay constant of Ca^2+^ transient) [17]. Changes in [Ca]_i_ in intact paced myocytes are expressed as an F/F_0_ where F represents cellular Fluo-4 fluorescence and F_0_ is diastolic Fluo-4 fluorescence. Ca^2+^ waves and Ca^2+^ spark frequencies were measured during a resting period upon stop of electrical stimulation at steady-state (i.e., a minimum of two minutes electrical stimulation) and manually quantified in a blinded fashion. Ca^2+^ wave propagation velocity was measured as previously described [19]. Ca^2+^ spark parameters were analyzed using the automated ImageJ Plugin SparkMaster [20]. SR Ca^2+^ content was assessed in longitudinal line scans after Ca^2+^ spark/wave measurements using Caffeine (20 mM) evoked transients [21]. Analysis of Ca^2+^ transients was performed using ImageJ and Liscana (IDL) [17].

### 2.4. Statistics

All data are presented as mean ± standard deviation. Data analysis was performed in a blinded fashion with respect to genotypes. GraphPad Prism was used for statistical inference and plotting (GraphPad Software, San Diego, CA, USA). To test for group differences, student’s *t*-test, Kruskal–Wallis One Way ANOVA on Ranks, or Chi-square test (dichotomous variables) was used. A *p* < 0.05 indicates significant statistical difference between groups.

## 3. Results

### 3.1. AF Inducibility in a Murine Model

**Figure 1:**
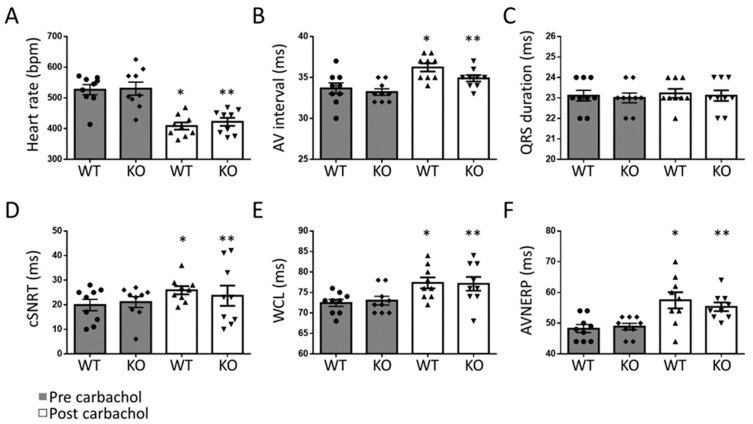
Electrophysiological properties in G_q_-KO (n = 11) and control mice (n = 10) before and after vagal stimulation with carbachol. Heart rate (**A**), atrioventricular (AV) interval (**B**), QRS duration (**C**), corrected sinus node recovery time (cSNRT, **D**), Wenckebach cycle length (WCL, **E**), and effective refractory period of the AV-node (AVNERP, **F**). * *p* < 0.05 vs. WT pre carbachol. ** *p* < 0.05 vs. KO pre carbachol.

**Figure 2:**
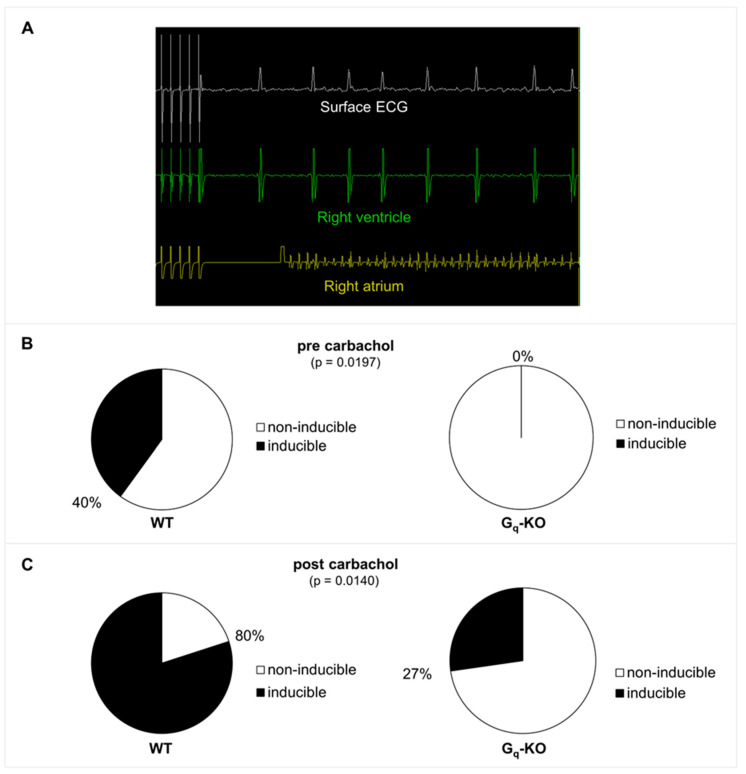
(**A**) Example of AF induction by programmed stimulation in a wildtype control mouse before carbachol injection. There is a short blanking period in the atrial channel following pacing. (**B**) AF inducibility in G_q_-KO (n = 11) versus control mice (n = 10) before application of carbachol. (**C**) AF inducibility 2 min after vagal stimulation with carbachol (intraperitoneal injection of 50 ng/g carbachol).

In order to define the role of G_q_-mediated signaling in AF in vivo, we used a transgenic mouse line with a tamoxifen-inducible, cardiomyocyte-specific Gα_q_/Gα_11_-deficiency (G_q_-KO) [14]. G_q_-KO and control mice underwent intracardiac electrophysiological studies using a 2F Octapolar catheter inserted via the right jugular vein. The inducibility of AF was determined before and after carbachol-induced vagal activation using a standardized protocol of programmed electrical stimulation [22]. Baseline electrophysiological parameters, including heart rate, sinus node recovery time, and atrial as well as AV nodal effective refractory periods, were comparable in G_q_/G_11_-KO vs. control mice, with no significant differences between the two groups before or after carbachol-induced vagal activation (Figure 1). While AF could be induced in four out of 10 control mice, it was not inducible in any of the 11 G_q_/G_11_-KO mice before carbachol administration (Figure 2A,B). Two minutes after vagal stimulation with 50 ng/g carbachol (i.p.), which resulted in a heart rate decrease of 15–20%, atrial pacing-induced AF in 8 out of 10 control mice (80%) but only in 3 out of 11 G_q_/G_11_-KO mice (27%) (Figure 2C). Moreover, the mean duration of AF episodes was significantly shorter in G_q_/G_11_-KO (23 ± 16 s) than in control mice (89 ± 14 s).

### 3.2. Baseline Characteristics of Excitation–Contraction Coupling in a Murine Model

**Figure 3:**
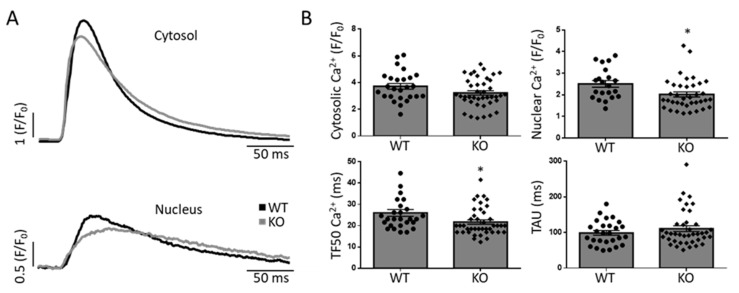
Properties of Ca^2+^ signaling during excitation–contraction coupling in a murine model. Example of cytosolic and nuclear Ca^2+^ transients during field-stimulation (**A**) and quantification (**B**) of maximal cytosolic and nuclear Ca^2+^ release, time to 50% of maximal cytosolic Ca^2+^ release (TF50) and the time constant of Ca^2+^ decay/removal (TAU). * *p* < 0.05 vs. WT. Each data point represents an independent cell preparation and experiment (Cytosolic: WT n = 26, KO n = 41; nuclear: WT n = 21, KO n = 37; TF50: WT n = 27, KO n = 41; TAU: WT n = 27, KO n = 40). The total number of animals per group was n = 5 (see Supplementary Figure S1 for per-animal analyses).

We studied excitation–contraction coupling in WT and G_q_-KO mice to elucidate further the role of the G_q_ pathway for pro-arrhythmogenic Ca^2+^ release. During electrical field stimulation, cytosolic Ca^2+^ transient amplitudes were unchanged upon G_q_ knockout (F/F_0_; 3.7 ± 0.2 vs. 3.3 ± 0.2 a.u. in G_q_-KO, n.s.). Diastolic Ca^2+^ removal measured by assessment of the time-constant TAU (monoexponential fit of the Ca^2+^ decay phase) was also unaltered (98 ± 7 vs. 111 ± 8 ms in G_q_-KO, n.s.). However, in G_q_-KO, time to 50% of maximal Ca^2+^ release was significantly shortened (26 ± 2 vs. 22 ± 1 ms in G_q_-KO, *p* < 0.05). In addition, nuclear Ca^2+^ release, as assessed by peak F/F_0_ within the nuclear compartment, was significantly reduced in atrial cardiomyocytes from G_q_-KO animals (F/F_0_; 2.5 ± 0.2 vs. 2.0 ± 0.1, *p* < 0.05; Figure 3 and Supplementary Figure S1).

### 3.3. Cellular and Subcellular Pro-Arrhythmogenic Ca^2+^ Release

**Figure 4:**
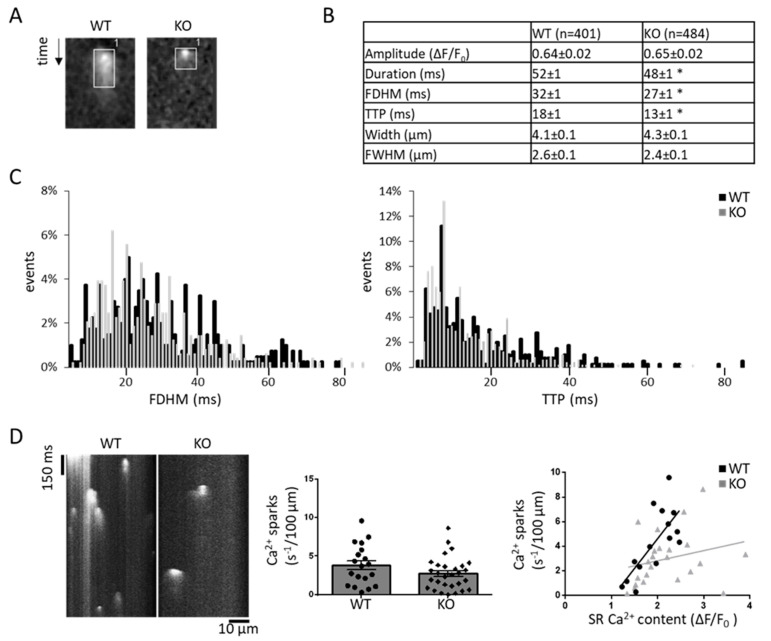
Subcellular Ca^2+^ spark properties in a murine model. Example and quantification of Ca^2+^ sparks in WT and G_q_-KO (**A**,**B**) as well as the distribution (**C**) of full duration at half maximum (FDHM) and time to peak (TTP) in all cells. Example for Ca^2+^ sparks in WT (n = 19) and G_q_-KO (n = 29) and their respective overall frequency without and with correlation to the sarcoplasmic reticulum Ca^2+^ content, respectively, (WT n = 15, KO n = 23) (**D**). * *p* < 0.05 vs. WT. Each data point represents an independent cell preparation and experiment. The total number of animals per group was n = 5 (see Supplementary Figure S1 for per-animal analyses).

Next, we tested the hypothesis of altered arrhythmogenic Ca^2+^ release at a (sub-) cellular level in G_q_-KO. To obtain subcellular Ca^2+^ release properties, we quantified individual spontaneous Ca^2+^ spark characteristics in WT and G_q_-KO (Figure 4, Supplementary Figure S1): Ca^2+^ sparks from G_q_-KO animals were of equal amplitude and width, yet significantly shorter and with a decreased time to peak Ca^2+^ release (Figure 4B). This finding becomes even more apparent using histogram analysis of the Ca^2+^ spark full duration at half maximum and time to peak (Figure 4C). Overall, Ca^2+^ spark frequency did not differ significantly (3.8 ± 0.6 vs. 2.7 ± 0.4 in G_q_-KO, *p* = 0.1); however, when corrected for SR Ca^2+^ content, Ca^2+^ sparks occurred less often in G_q_-KO at a given Ca^2+^ content as compared to WT. In addition, with increasing SR Ca^2+^ content, the observed increase of Ca^2+^ spark frequency was less pronounced in G_q_-KO as compared to WT (Figure 4D and Supplementary Figure S2).

**Figure 5:**
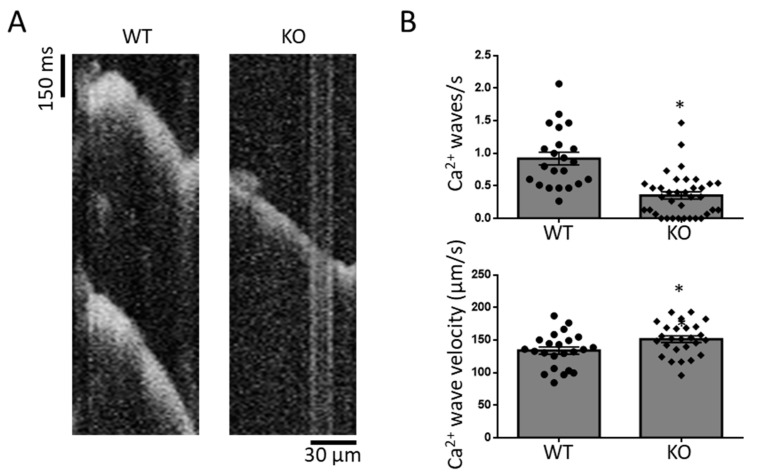
Arrhythmogenic Ca^2+^ waves in WT and G_q_-KO mice (**A**). Ca^2+^ wave frequency and propagation velocity (**B**). * *p* < 0.05 vs. WT. Each data point represents an independent cell preparation and experiment (Ca^2+^ wave frequency: WT n = 22, KO n = 36; Ca^2+^ wave velocity: WT n = 22, KO n = 26). The total number of animals per group was n = 5 (see Supplementary Figure S1 for per-animal analyses).

In line with this notion, we finally assessed cellular pro-arrhythmogenic Ca^2+^ wave activity: Ca^2+^ wave frequency was significantly reduced in G_q_-KO compared to WT. Of note, G_q_-KO also significantly altered Ca^2+^ wave propagation velocity in longitudinal line scans (Figure 5 and Supplementary Figure S1).

## 4. Discussion

Here we elucidate the central role of arrhythmogenic G_q_-mediated signaling in the pathomechanism of AF. Cardiomyocyte-specific inactivation of G_q_ significantly reduced AF inducibility in a murine AF model. Our in vitro data in left atrial cardiomyocytes from G_q_-KO mice indicate fewer spontaneous Ca^2+^ waves and altered Ca^2+^ spark properties as a potential mechanism of action.

### 4.1. Targeting Arrhythmogenic G_q_-Signaling with G_q_-Coupled Receptor Antagonists

G_q_-signaling via IP_3_R is initiated by G_q_-coupled receptors. Arrhythmogenic effects have been consistently demonstrated for the predominant cardiac G_q_-coupled receptors, namely the angiotensin II receptor type 1 (AT1 receptor), the endothelin-1 receptor A (ETA receptor), the M3 muscarinic acetylcholine receptor (M3 receptor), thrombin (PAR) receptors and the alpha-1 adrenergic receptor [4]. In particular, abundant data implicate AT1 receptors in the pathogenesis of AF, and their inhibition has been shown to protect from AF in numerous animal models [23].

While arrhythmogenic effects have in part been attributed to G_q_-mediated profibrotic and proinflammatory signaling [4,24], direct proarrhythmogenic effects of AT1- and ETA-receptors have been increasingly appreciated in recent years [25,26]. In this regard, both angiotensin II-receptor type 1 and ET-1 receptors have been shown to enhance ectopic activity by promoting Ca^2+^ leak and delayed after-depolarisations [10,25,26].

However, while indirect evidence from numerous clinical trials indicated that chronic inhibition of AT1 receptor signaling significantly reduces the incidence of AF, large randomized trials failed to demonstrate the beneficial effects of AT1 antagonists on AF [27,28,29]. Against this background, it has to be considered that conventional AT1 antagonists do not selectively inhibit G_q_-mediated signaling but equally block all downstream signaling pathways, some of which may even have beneficial effects. Thus, selective inhibition of G_q_-mediated signaling may be desirable. This could be accomplished by biased ligands that act as G_q_-selective antagonists. In fact, we have recently identified biased AT1-ligands that selectively inhibit G_q_-mediated signaling (unpublished data). This angiotensin analog (TRV027) has proven to be well-tolerated and safe in phase II clinical trials in the context of heart failure. It may thus qualify as a suitable candidate for a G_q_-targeting AF therapy [30].

### 4.2. G_q_-Dependent Mechanoelectrical Feedback

While many humoral stimuli can activate G_q_-signaling through G-protein-coupled receptors, we have recently shown that this pathway is also mechanosensitive [6]. Mechanical stretch is a well-established determinant of atrial size and function, and immediate as well as chronic responses to mechanical forces can promote arrhythmia [5]. However, the molecular mechanisms that link mechanical forces to arrhythmogenesis are incompletely understood. The recent discovery of the mechanosensitive non-selective cation channel Piezo1 was a breakthrough in the field of mechanotransduction [31]. However, even though Piezo channels are expressed in the heart and have been implicated in cardiac arrhythmia, evidence of their cardiac function is still sparse.

Interestingly, we recently demonstrated that Piezo1 and G_q_ form a functional mechanosignaling complex in endothelial cells that may also be operative in cardiomyocytes [6]. This complex regulates IP_3_R-mediated Ca^2+^-signaling as well as NFκB-mediated proinflammatory signaling in response to mechanical forces—both key processes in the pathogenesis of AF. Thus, it is intriguing to speculate that G_q_-mediated mechanotransduction is also involved in the arrhythmogenic mechanoelectrical feedback in the context of AF.

### 4.3. G_q_-Signaling and Vagally-Dependent Atrial Fibrillation

Vagotonic conditions are known to promote AF, and in some patients, AF episodes are clearly vagally dependent [32,33]. Acetylcholine released by vagal nerve endings has been shown to stimulate G_i_-coupled M2 muscarinergic receptors that activate G-protein- gated K+ channels, thereby reducing atrial action potential duration and increasing susceptibility to early after-depolarisations as well as reentrant mechanisms [34]. However, while arrhythmogenic effects have been largely attributed to G_i_-mediated signaling downstream of M2 muscarinic receptors, G_q_-coupled M3 muscarinic receptors are also expressed in the atria and appear to mediate arrhythmogenic effects of vagal activation to some extent [35,36]. These arrhythmogenic M3 effects seem to involve DADs generated by abnormal Ca^2+^ events. Thus, in light of the data presented here, the relative contribution of G_i_-coupled M2 vs. G_q_-coupled M3 receptors to vagally dependent AF may have to be reconsidered.

### 4.4. The Role of the G_q_ Pathway for SR Ca^2+^ Leak and Pro-Arrhythmogenic Cellular Conditions

IP_3_R-mediated Ca^2+^ release has been shown to also facilitate SR Ca^2+^ release via sensitization of nearby RyR clusters [21]. Atrial cardiomyocytes from G_q_-KO animals showed decreased spontaneous SR Ca^2+^ release in support of this notion. Interestingly, cytosolic Ca^2+^ release during excitation–contraction coupling, i.e., Ca^2+^ transient amplitude, was not significantly affected by G_q_ knockout, indicating unaltered baseline Ca^2+^ signaling. Of note, spontaneous Ca^2+^ release events were reported to be increased in human atrial cardiomyocytes during chronic AF, and enhanced SR Ca^2+^ leak has been associated with DADs in this setting [7]. Normalizing SR Ca^2+^ leak, e.g., through genetic inhibition of Ca^2+^/calmodulin-dependent protein kinase II-mediated (CaMKII) RyR2-S2814 phosphorylation, was shown to delay the development of spontaneous atrial ectopy and fully prevent AF in mice [37]. G_q_-mediated signaling altering SR Ca^2+^ release might represent another piece of the puzzle of IP_3_R-dependent SR Ca^2+^ leak causally linked to the development of AF.

As the use of all currently approved antiarrhythmic drugs is limited by potentially life-threatening ventricular pro-arrhythmic effects, from a translational perspective, it is intriguing that IP_3_R expression in atrial myocytes is 6- to 10-fold higher than in ventricular myocytes [13,38], and that IP_3_R-mediated electrophysiological effects on Ca^2+^ homeostasis are absent in ventricular myocytes [12].

We also report a decrease of nuclear Ca^2+^ transient amplitudes upon G_q_ knockout, further underscoring the notion of abundant IP_3_R expression in the nuclear envelope [19]. These findings have important ramifications in the setting of AF: Only recently has AF been shown to increase atrial-cardiomyocyte nucleoplasmic Ca^2+^ by IP_3_R-upregulation, leading to enhanced IP_3_R-CaMKII-HDAC4 signaling and L-type calcium current downregulation [39]. Altered nuclear Ca^2+^, mediated via the G_q_ signaling cascade, might, therefore, directly affect gene regulation important for Ca^2+^ release and SR Ca^2+^ leak in the setting of AF.

### 4.5. Altered Calcium Handling as an Arrhythmogenic Substrate

The impact of increased calcium release from the sarcoplasmic reticulum on AF inducibility in vivo may be counterintuitive, as it has primarily been regarded as a mechanism of triggered activity-inducing AF rather than an arrhythmogenic substrate sustaining AF. However, our findings are in line with a number of previous studies in which diastolic calcium leak from the sarcoplasmic reticulum was not associated with triggered activity resulting in spontaneous AF events but with an increased AF inducibility by programmed stimulation in mice [40,41]. Taken together, these data indicate that altered calcium release can create an arrhythmogenic substrate favoring AF initiation and maintenance.

## 5. Conclusions

Our combined in vitro and in vivo studies in mice with cardiomyocyte-specific G_q_-deficiency demonstrate that G_q_-mediated signal transduction promotes arrhythmogenic Ca^2+^-release and AF in mice. These data suggest that G_q_-signaling likely mediates arrhythmogenic effects of angiotensin II, vagal stimulation, mechanical stress, and other stimuli known to promote AF. Thus, targeting the G_q_-pathway, ideally using G_q_-selective biased receptor ligands, may be a promising approach for the treatment and prevention of AF. Importantly, the atrial-selective expression of the G_q_-effector IP_3_R confers atrial selectivity mitigating the risk of life-threatening ventricular pro-arrhythmic effects.

## Data Availability

The data underlying this article will be shared upon reasonable request to the corresponding author.

## Supplementary Materials

The following supporting information can be downloaded at: https://www.mdpi.com/article/10.3390/biomedicines11020526/s1, Figure S1: Per-animal analyses of Ca^2+^ signaling. Figure S2: SR Ca_2+_ content in WT and KO mice as obtained with caffeine (top).
