# Supplementary material for: Gq-Mediated Arrhythmogenic Signaling Promotes Atrial Fibrillation
Source: Biomedicines. 2023 Feb 11;11(2):526. doi: 10.3390/biomedicines11020526 (PMC9953645; doi:10.3390/biomedicines11020526)
Supplement: Supplementary file 1 [file biomedicines-11-00526-s001.zip › biomedicines-2213123-supplementary.pdf]

G_q-mediated arrhythmogenic signaling promotes atrial fibrillation

Felix Hohendanner, M.D. Ph.D.[1,2,3]; Ashok Prabhu[1], Nicola Wilck, M.D.[2,3,4,5,6]; Verena Stangl, M.D. [2,3,7]; Burkert Pieske, M.D.[1,2,3]; Karl Stangl, M.D.[2,3,7]; Till F. Althoff, M.D.[2,3,7,8,9]

[1] Charité – University Medicine Berlin, Campus Virchow-Klinikum, Department of Cardiology and German Heart Center, Augustenburger Platz 1, 13353 Berlin, Germany

[2] DZHK (German Centre for Cardiovascular Research), partner site Berlin, 13316 Berlin, Germany

[3] Berlin Institute of Health at Charité – Universitätsmedizin Berlin, 10117 Berlin, Germany

[4] Max Delbrück Center for Molecular Medicine in the Helmholtz Association (MDC), 13125 Berlin, Germany

[5] Experimental and Clinical Research Center (ECRC), a cooperation of Charité - Universitätsmedizin Berlin and Max Delbruck Center for Molecular Medicine (MDC), 13125 Berlin, Germany

[6] Department of Nephrology and Medical Intensive Care Medicine, Charité - Universitätsmedizin Berlin, 10117 Berlin, Germany

[7] Charité – University Medicine Berlin, Department of Cardiology and Angiology, Charité Campus Mitte, Charitéplatz 1, 10117 Berlin, Germany

[8] Arrhythmia Section, Cardiovascular Institute (ICCV), Hospital Clínic, Universitat de Barcelona, C/Villarroel N° 170, 08036 Barcelona, Catalonia, Spain

[9] Institut d'Investigacions Biomèdiques August Pi i Sunyer (IDIBAPS), 08036 Barcelona, Catalonia, Spain

Correspondence: althoff@clinic.cat

Short Title: G_q-signaling in atrial fibrillation

Supplementary Figures

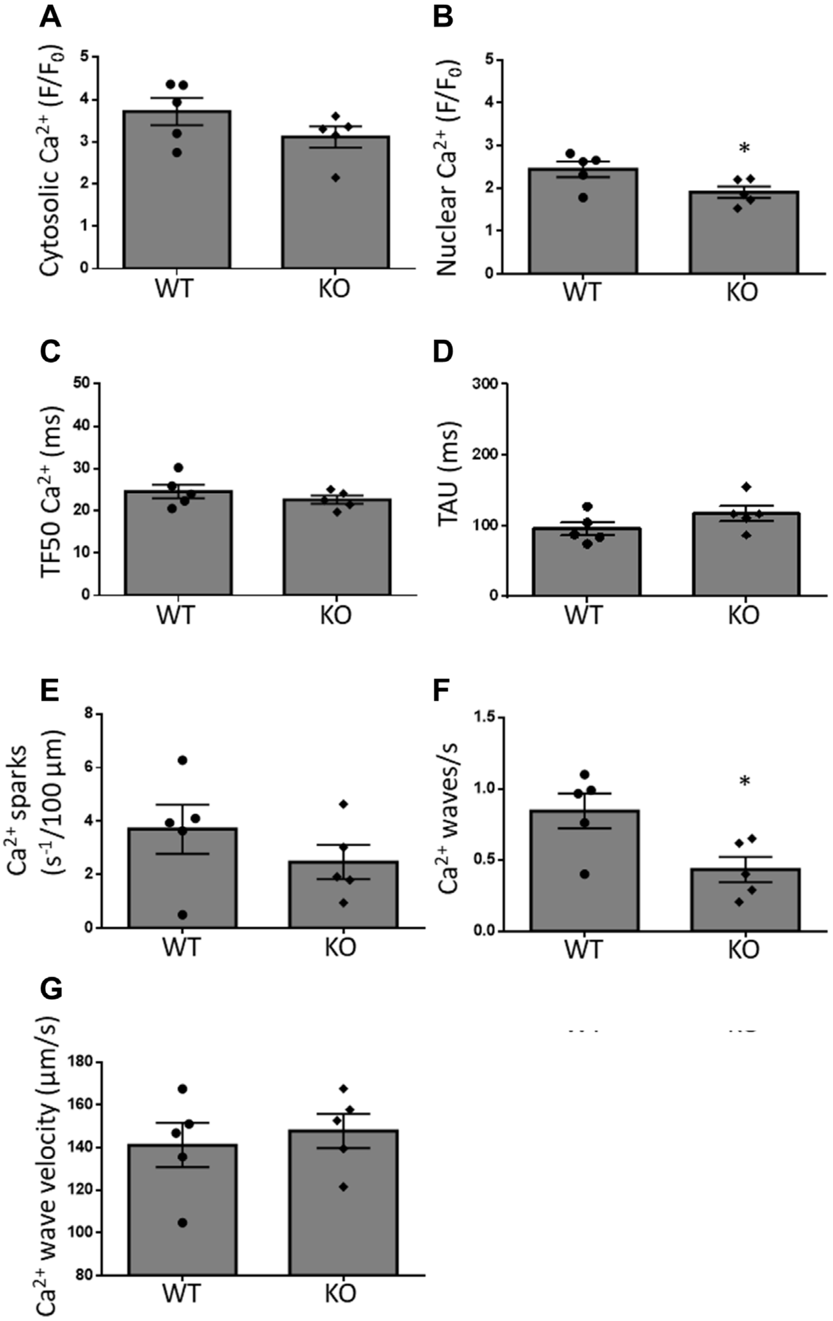

Supplementary Figure S1. Per-animal analyses of Ca^{2+} signaling. Quantification of maximal cytosolic (A) and nuclear Ca^{2+} release (B), time to 50% of maximal cytosolic Ca^{2+} release (TF50) (C), the time constant of Ca^{2+} decay/removal (TAU) (D), subcellular Ca^{2+} spark frequency (E), as well as arrhythmogenic Ca^{2+} wave frequency (F) and propagation velocity (G). The total number of animals per group was n=5. *p<0.05 vs. WT.

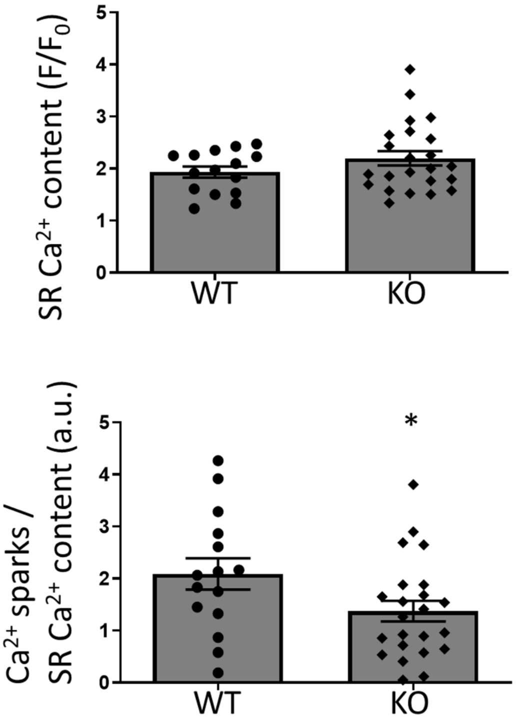

Supplementary Figure S2. SR Ca2+ content in WT and KO mice as obtained with caffeine (top). Ca2+ spark frequency as corrected for SR Ca2+ content (ratio, a.u.). *p<0.05 vs. KO.
